# Measurement of the apical anatomy of immature maxillary central incisors using cone-beam computed tomography

**DOI:** 10.1186/s12903-023-03569-9

**Published:** 2023-11-07

**Authors:** Linlin Wang, Xiurong Qin, Yongzhi Pang, Yuxin Ma, Mingru Fan, Hongsheng Tian

**Affiliations:** 1 Medical imaging department, Jinan Stomatological HospitalSong, Jinan, 250001 China; 2 Pediatric dentistry department, Jinan Stomatological Hospital, Jinan, 250001 China; 3 Oral and Maxillofacial Surgery, Jinan Stomatological Hospital, Jinan, 250001 China; 4 Medical imaging department, Shandong University of Traditional Chinese Medicine, Jinan, 250011 China; 5 Shandong University of Traditional Chinese Medicine, Wenhua Road NO 42, Jinan, 250011 Shandong Province China

**Keywords:** Incisor, Anatomy, Apical foramen, Cone-beam computed tomography

## Abstract

**Objective:**

To investigate the CBCT findings of the apical anatomy of immature maxillary central incisors.

**Methods:**

CBCT images of 100 immature maxillary central incisors in Nolla 8 and 100 immature maxillary central incisors in Nolla 9 were collected. The mesiodistal and carniocaudal diameters of the apical foramen of immature maxillary central incisors were measured by software included with CBCT, as well as the mesiodistal, carniocaudal and facioligual diameters of the apical shadow. The apical shadow and apical foramen diameters were compared between Nolla 8 and Nolla 9. Data were analyzed using the MedCalc software package.

**Results:**

For immature maxillary central incisors, the mesiodistal and facioligual diameters of the apical foramen were 2.75±0.68 mm and 3.28±0.74 mm in Nolla 8 and 1.50±0.51 mm and 1.92±0.79 mm in Nolla 9. The mesiodistal, facioligual and carniocaudal diameters of the apical shadow were 3.84±0.73 mm, 4.49±0.68 mm and 3.41±1.27 mm in Nolla 8 and 2.76±0.60 mm, 3.41±0.80 mm and 2.06±0.65 mm in Nolla 9, respectively.

**Conclusions:**

The immature maxillary central incisors in Nolla 8 have a larger apical shadow and apical foramen than those in Nolla 9. The apical region of the maxillary central incisors in Nolla 8 was more likely to have a broad, blurred lamina dura. With the development of the apical foramen, the lamina dura in the apical region tended to be clear and sharp.

**Clinical significance:**

To our knowledge, this is the first study to radiologically analyse the in vivo anatomy of the apical foramen and apical shadow of immature maxillary central incisors. The results of this study provide a more detailed understanding of the apical anatomy of the immature maxillary central incisor for the diagnosis and treatment of apical lesions.

In 1960, Nolla [1] published a method for classifying the stages of dental development using radiographs. It divides dental development into 10 stages and is now widely used in clinical practice. The earliest eruption of the maxillary central incisor is seen at the age of 5, with peak eruption at age 6–8. It takes approximately 2 or 3 years from eruption to completion of root development, and the root length is usually 2/3 to 3/4 of the final root length when the tooth emerges from the gingiva. According to the Nolla staging method, the teeth at this stage are mostly in Nolla 8 and Nolla 9 [2], and the teeth at this stage are called immature teeth. Immature teeth are characterized by incomplete root development, an unclosed apical foramen, and residual papilla and dental sac in the apical region. On X-ray film, it usually appears as a low-attenuation lesion with circumscribed margins. Periapical periodontitis of immature teeth is one of the most common diseases in paediatric dentistry. It also presents as a low-attenuation lesion and the lamina dura may be partially or completely destroyed. Simultaneously, the majority of traumatized teeth are immature teeth [3]. Among them, maxillary central incisors have the highest trauma rate [4, 5]. When pulp necrosis occurs in maxillary central incisors, root development is arrested and he apical foramen is widened [6]. Therefore, it is important to understand the anatomical characteristics of the apical region of immature maxillary central incisors in Nolla 8 and Nolla 9 in order to identify apical injuries or lesions. At different stages of development, the sizes of the apical foramen and apical shadow were different. The dimensions of the apical foramen and the apical shadow of immature maxillary central incisors in Nolla 8 and Nolla 9 are not known, nevertheless.

CBCT has been widely used in oral clinics since the 1990s. It has isotropic characteristics and can image the anatomical structure 1:1. Haridas [7] noted that CBCT is a reliable and nondestructive method in vivo that can be used commonly to measure the anatomical structure of teeth. Therefore, the aim of this study is to describe the dimensions of the apical shadow and apical foramina to provide a reference for the identification of apical lesions.

## Materials and methods

### Study setting and participants

The trial protocol was approved by the institutional review board of the hospital (No. 20,190,104). Children who underwent CBCT in our imaging department between January 2020 and December 2020 were selected. The CBCT images were mainly taken for the purposes of orthodontic treatment, dental caries, maxillofacial tumour and cyst. Inclusion criteria were healthy immature maxillary central incisors, an undeveloped apical foramen, aged from 6 to 9 years, good image quality without artifacts. After statistics, 778 cases were included. Exclusion criteria were supernumerary teeth in maxillary anterior region, crowded dentition in maxillary anterior region, maxillofacial tumour and cyst in maxillary anterior region. After evaluation, 269 cases were excluded from 778. Two paediatric dental specialists who were blinded to the clinical and CBCT information, classified immature maxillary central incisors into Nolla 8 and Nolla 9 according to the Nolla staging system. Furthermore, We measured the unilateral immature maxillary central incisors in random order to avoid selection bias. The sample size was calculated by the MedCalc software package. In order to accurately determine the sample size, we conducted a pre-experiment because we could not find a similar previous study. In this pre-experiment, we measured the mesiodistal diameters of the apical shadows of 10 immature maxillary central incisors in Nolla 8 and 10 in Nolla 9. Considering the mean and standard deviation of the obtained in the pre-experiment, the following parameters were used in the design: Alpha (α=5%), Beta (β=10%), average difference (µ_d_=1), Nolla 8 and Nolla 9 standard deviations (σ=1.2 and 0.8 respectively), and sample rate (= 1). In total, 46 should be recruited. However, the sample size was increased to 100 to be adequate for achieving objectives of the study. Stratified sampling method was used to acquire the final study group. Finally, 100 immature maxillary central incisors in Nolla 8 and 100 in Nolla 9 from 509 patients were evaluated in this study. Written informed consent was obtained from all guardians of the participants.

### Methods

**Fig. 1:**
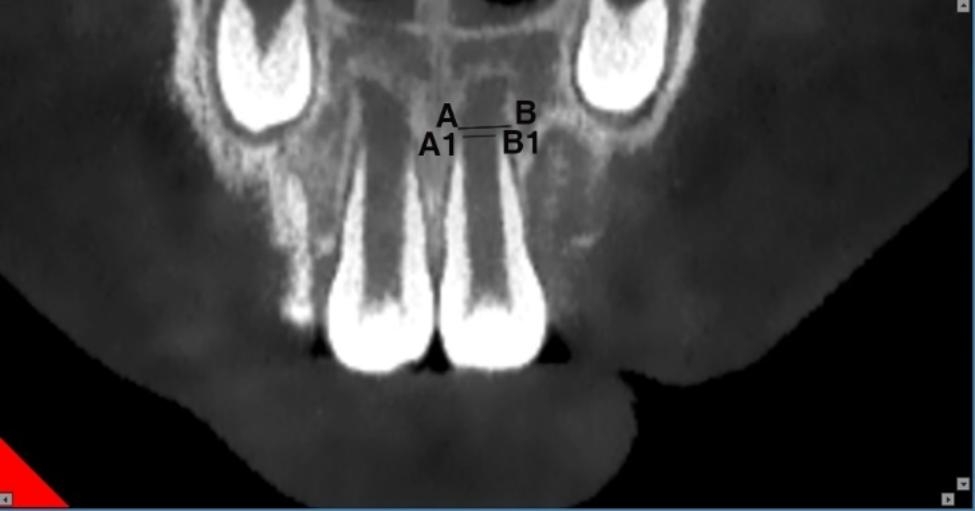
Coronal plane of the immature maxillary central incisor. AB, The mesiodistal diameter of the apical shadow of the immature maxillary central incisor; A1B1, The mesiodistal diameter of the apical foramen of the immature maxillary central incisor

**Fig. 2:**
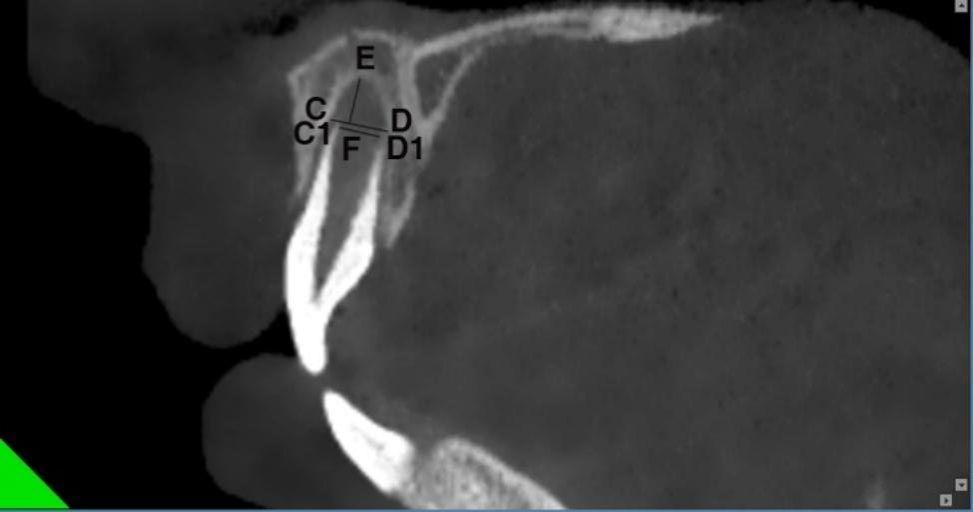
Sagittal plane of the immature maxillary central incisor. CD, facioligual diameter of the apical shadow of the immature maxillary central incisor; C1D1, facioligual diameter of the apical foramen of the immature maxillary central incisor; EF, carniocaudal diameter of the apical shadow of the immature maxillary central incisor

All examinations were performed on a New Tom 5G CBCT scanner (QR Verona, Verona, Italy). Images were reformatted to a horizontal line parallel to the incisal edge and a sagittal line parallel to the long axis of the maxillary central incisor by software MPR included with CBCT. While maintaining this interface, the mouse was then moved until a maximum cross-section of the apical shadow of the immature maxillary central incisor was obtained in the coronal planes. Measurements were then taken in the coronal plane, including the mesiodistal diameters of the apical foramen and the apical shadow (Fig. 1). The maximum cross-section of the apical shadow of the immature maxillary central incisor in the sagittal planes was obtained using the same method. and then the facioligual diameter of the apical foramen and apical shadow and the carniocaudal diameter of the apical shadow were measured (Fig. 2). The morphological characteristics of the lamina dura in the apical region were also observed. All measurements were made by a blinded experienced radiologist. The measurements were repeated once at one-week intervals, and the mean values were taken for statistical analysis.

### Consistency test

Ten teeth (5 each in Nolla 8 and Nolla 9) were randomly selected for measurement by the same investigator, and 10 sets of data were obtained. Two weeks later, the second measurements were taken on the 10 teeth, and another 10 sets of data were obtained. Interclass correlation coefficients and Bland‒Altman plots were used to assess the reliability of the first and second measurements.

### Statistical analysis

All analyses were performed with the MedCalc software package. The Shapiro‒Wilk test was conducted to test data distribution. Normally distributed data of continuous variables are presented as the mean±SD, and categorical data are presented as n (%). Data were compared using the parametric *t* test for normally distributed data and the *χ*^2^ test for categorical data. Similarly, the Pearson correlation test was used to analyze the correlation between the diameters of the apical shadow and the apical foramen. Regression equations for the carniocaudal diameter of the apical shadow and the mesiodistal diameter of the apical foramen were established. P values < 0.05 were considered significant.

## Results

### Characteristics of participants

The institutional review board of the hospital. Imaging data of 200 immature maxillary central incisors were included in the PACS system of our imaging department. Baseline characteristics are shown in Table 1.

**Table 1:** Comparison of baseline characteristics between Nolla 8 and Nolla 9

Characteristic	Total	Nolla 8	Nolla 9	Value	*P*
continuous variable‾*x* ± s				*t*	*t* test
Age(year)	7.88±0.82	7.36±0.59	8.40±0.67	11.64	0.00
Male	7.84±0.80	7.32±0.47	8.35±0.72	8.55	0.00
Female	7.92±0.84	7.40±0.70	8.45±0.61	7.92	0.00
*t*	0.67	0.67	0.72		
*P*	0.50	0.50	0.47		
categorical data %				*χ* ^*2*^	*χ*^2^ test
Male	50.5%(101/200)	50%(50/100)	51%(51/100)		
Female	49.5%(99/200)	50%(50/100)	49%(49/100)	0.02	1.00

### Consistency check

**Fig. 3:**
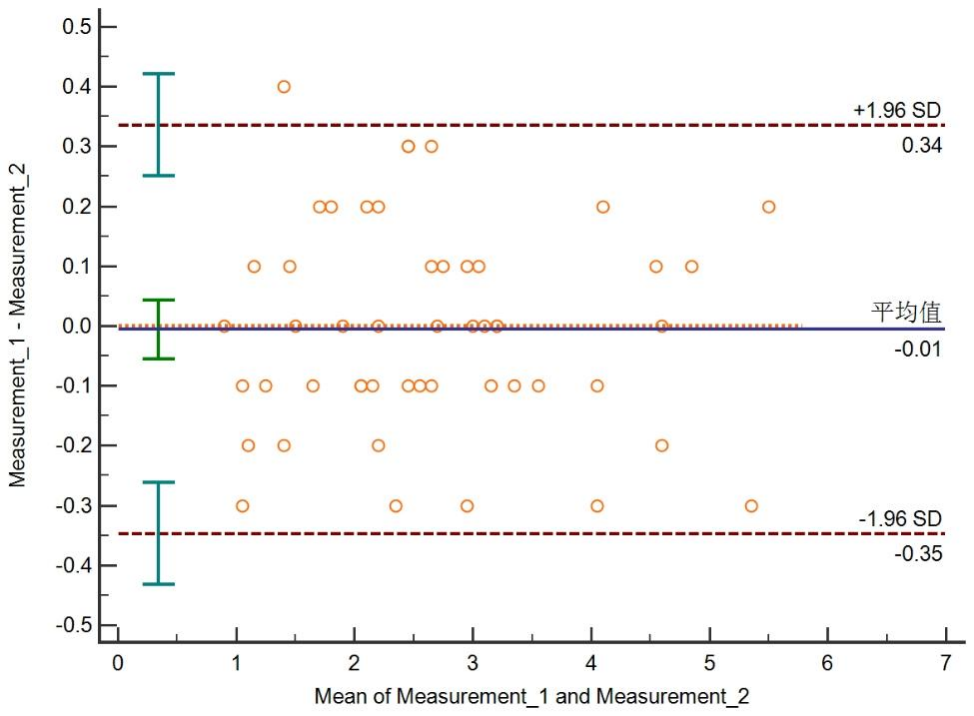
Bland‒Altman plots for Measurement 1 and Measurement 2

Bland‒Altman plots of 10 paired data points for the first and second measurements are shown in Fig. 3, and 2% of the points were outside the 95% consistency boundary. 98% of the points were within the 95% limits. The maximum absolute value of the difference was 0.4, and the average difference between the two results was −0.01, which is clinically acceptable for such a small margin; therefore, the two measurements could be considered to have excellent consistency. The interclass correlation coefficient also showed excellent consistency for the first and second measurements (ICC = 0.986).

### Morphology of the lamina dura in the apical region

**Fig. 4:**
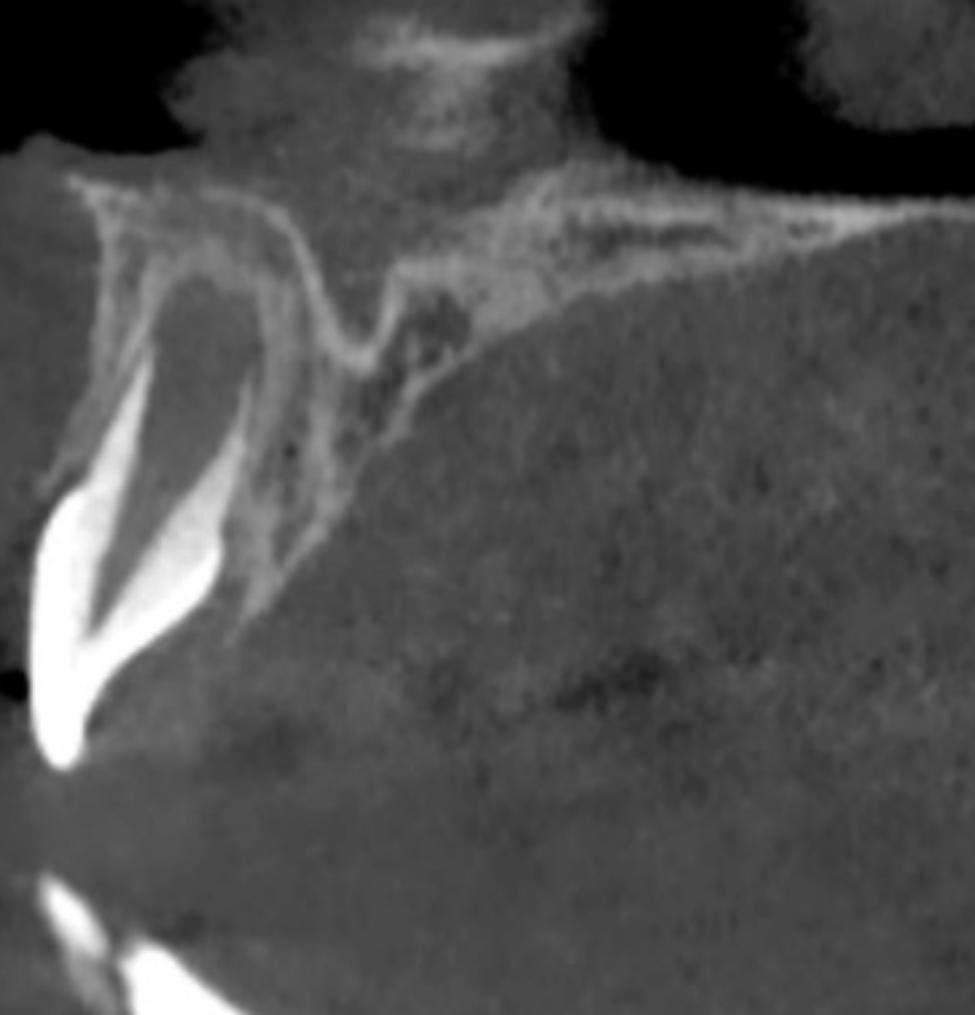
Lamina dura of maxillary central incisor in Nolla 8

**Fig. 5:**
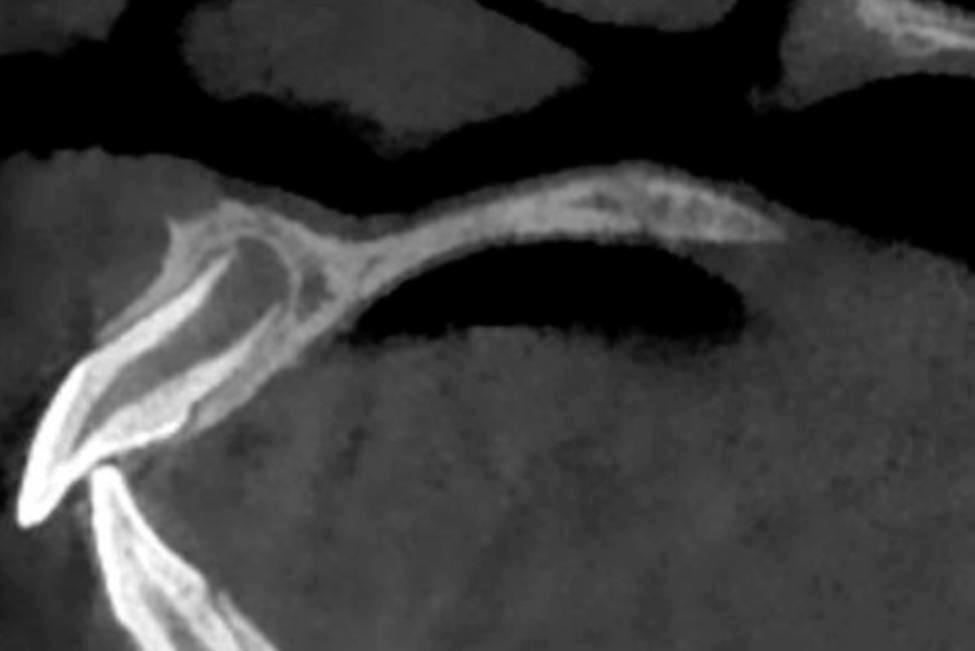
Lamina dura of maxillary central incisor in Nolla 9

In the apical region of maxillary central incisors in Nolla 8, the lamina dura was more likely to be broad and blurred, and with the development of the apical foramen, the lamina dura in the apical region tended to be clear and sharp, as shown in Figs. 4 and 5.

### The measured results

The results showed that the diameters of the apical shadow and apical foramen of immature maxillary central incisors conformed to a normal distribution. The results of the diameters of apical shadow and apical foramen of immature maxillary central incisors in Nolla 8 and Nolla 9 are shown in Table 2. Immature maxillary central incisors in Nolla 8 showed significantly larger apical shadow and apical foramen than those in Nolla 9 (*P* < 0.05), as shown in Table 2. The results of the diameters of the apical shadow and apical foramen in different sexes are shown in Table 3. Except for the facioligual diameter of the apical foramen in Nolla 8 (*P* > 0.05), each diameter of the apical shadow and apical foramen of immature maxillary central incisors was significantly larger in males than in females (*P* < 0.05). The results are presented in Table 3.

According to the correlation analysis, the mesiodistal, facioligual and carniocaudal diameters of the apical shadow were positively correlated with the mesiodistal and facioligual diameters of the apical foramen (*P* < 0.001), and the regression equation is shown in Table 4.

**Table 2:** Comparison of apical shadow and apical foramen diameters between Nolla 8 and Nolla 9 mm,‾*x* ± s

Parameter	Nolla 8	Nolla 9	*t*	*P*
Mesiodistal diameter of apical foramen	2.75±0.68	1.50±0.51	14.752	0.000
Facioligual diameter of apical foramen	3.28±0.74	1.92±0.79	12.646	0.000
Mesiodistal diameter of apical shadow	3.84±0.73	2.76±0.60	11.416	0.000
Facioligual diameter of apical shadow	4.49±0.68	3.41±0.80	10.271	0.000
Carniocaudal diameter of apical shadow	3.41±1.27	2.06±0.65	9.361	0.000

**Table 3:** Comparison of apical shadow and apical foramen diameters in different sexes mm,‾*x* ± s

Parameter	Nolla 8		*t*	*P*	Nolla 9		*t*	*P*
	Males	Females			Males	Females		
Mesiodistal diameter of apical foramen	2.92±0.75	2.59±0.57	2.442	0.016	1.68±0.54	1.32±0.39	3.855	0.000
Facioligual diameter of apical foramen	3.34±0.72	3.22±0.77	0.821	0.414	2.22±0.84	1.60±0.58	4.204	0.000
Mesiodistal diameter of apical shadow	4.06±0.82	3.61±0.55	3.214	0.002	2.91±0.69	2.60±0.44	2.721	0.007
Facioligual diameter of apical shadow	4.65±0.68	4.32±0.64	2.445	0.017	3.66±0.82	3.15±0.69	3.333	0.001
Carniocaudal diameter of apical shadow	3.98±1.35	2.85±0.93	4.869	0.000	2.21±0.64	1.91±0.63	2.384	0.019

**Table 4:** Correlation analysis of apical shadow and apical foramen diameters in immature maxillary central incisors

Variables		Mesiodistal diameter of apical shadow	Facioligual diameter of apical shadow	Carniocaudal diameter of apical shadow
Mesiodistal diameter of apical foramen	r^#^	0.90	0.70	0.55
	*P*	0.00	0.00	0.00
Facioligual diameter of apical foramen	r^#^	0.74	0.88	0.45
	*P*	0.00	0.00	0.00

#: Pearson correlation analysis

## Discussion

Periapical disease is one of the most frequent oral diseases. It may induce pathological changes in dental-pulp tissue, thereby affecting the prognosis and long-term preservation of the affected teeth. In particular, immature permanent teeth have the unique anatomical and physiological characteristics of incomplete root development, a large apical foramen and inadequate blood supply. Once immature permanent teeth become affected by periapical disease, treatment of the affected teeth becomes very challenging. When periapical disease occurs in immature permanent teeth, root development is arrested, the apical foramen is widened, and the lamina dura may be partially or completely destroyed [8]. The diagnosis of apical periodontitis is based on clinical and radiographic information, and immature teeth lack a reliable and predictable response to temperature and nuclear tests. Therefore, radiological assessment is essential both for making the correct initial diagnosis and for evaluating the outcome of treatment. [9]. It is helpful to distinguish periapical disease and observe root development by comparing the radiographic characteristics of the apical region during normal development.

To our knowledge, the immature maxillary central incisor is the earliest eruption of permanent teeth but is also the most prone to dental trauma of the permanent teeth [4]. When luxation of bilateral immature maxillary central incisors occurs, or when the degree of eruption of bilateral immature maxillary central incisors is different and luxation of one immature maxillary central incisor occurs, the degree of luxation cannot be assessed by comparison of bilateral immature maxillary central incisors. The measured data can be useful in determining the location for replantation of dislocated teeth and in guiding the decision for orthodontic traction and surgical reduction.

In clinical practice, digital periapical radiogram continues to be one of the most commonly used methods for periapical diseases [10]. It is relatively inexpensive, widely available and has no radiation burden, but two-dimensional overlap and distortion influence the accuracy of investigations [11, 12]. CBCT is advanced equipment used in oral medicine that provides three-dimensional images by a 180 to 360° rotation and offers a lower exposure in comparison with CT. Anatomical assessment using CBCT supplements the date of apical shadow and apical foramen and provides important information regarding clinical diagnosis and treatment.

The apical foramen of immature permanent teeth was not completely formed, and the apical foramen tended to close with age. In the current study, we demonstrated that the age in Nolla 8 was lower than that in Nolla 9. Our results support previous conclusions in children that the apical foramen is larger in Nolla 8 than in Nolla 9. Jiang [13] et al. used CBCT to study the apical structure of the mandibular second premolars in Nolla 8. The buccolingual diameters of the apical foramen were 3.86±0.96 mm and 3.51±0.95 mm, and the mesiodistal diameters of the apical foramen were 2.23±0.50 mm and 2.16±0.56 mm in males and females, respectively. Our results showed that the facioligual diameters of the apical foramina of immature maxillary central incisors in Nolla 8 were 3.34±0.72 mm in males and 3.22±0.77 mm in females, and the mesiodistal diameters of the apical foramina were 2.92±0.75 mm in males and 2.59±0.57 mm in females. Despite the different tooth positions, the apical foramen is similar in size. It is inferred that the size of the apical foramen in Nolla 8 may be similar in different tooth positions, and the specific results need to be verified in an in-depth study with an expanded sample size. Moreover, our present study demonstrated that a larger apical foramen of immature maxillary central incisors was detected in males than in females. Zhang [14] et al. found the eruption time of permanent teeth in girls earlier than in boys, which is a side-confirmation of the reliability of our study.

The periodontal development of immature permanent teeth was incomplete, and there were residual dental papilla and dental sacs in the apical region. Kim [15] et al. mentioned that the histological characteristics of dental papilla are oval mucous tissue 1.5 cm or less in diameter. In this retrospective study, we found that the apical shadow was greater in Nolla 8 than in Nolla 9. Basrani [2] noted that the residual papilla and papillary sac in the apical region show a low-attenuation lesion with circumscribed margins on radiographs. As the root develops, the low-attenuation lesion diminishes. As the periodontal tissue develops, the transmission image disappears. The apical region of the maxillary central incisors in Nolla 8 was more likely to have a broad, blurred lamina dura, and with the development of the apical foramen, the lamina dura in the apical region tended to be clear and sharp, which could be explained by the formation of dental cementum during tooth development. However, the specific mechanism remains to be investigated.

Our study had several limitations. First, due to the retrospective single-center design, sampling bias cannot be discounted. .A multicenter approach with a larger sample size is required to re-evaluate the results of our study. Second, the lack of a uniform standard for the morphology of the lamina dura could be underestimated its potential value in predicting periapical disease.

In conclusion, the clinical diagnosis and treatment of periapical periodontitis and lesions could be improved with a better understanding of apical anatomy.

## Data Availability

The datasets used and/or analyzed during the current study are available from the corresponding author on reasonable request.

## Abbreviations

CBCT: Cone-beam Computed Tomography
